# Associations between acute and chronic lifetime stressors and psychosis-risk symptoms in individuals with 22q11.2 copy number variants

**DOI:** 10.1017/S0033291723000740

**Published:** 2023-11

**Authors:** Jasmine Modasi, Vahe Khachadourian, Kathleen O'Hora, Leila Kushan, George M. Slavich, Grant S. Shields, Eva Velthorst, Carrie E. Bearden

**Affiliations:** 1 Department of Psychiatry, Icahn School of Medicine at Mount Sinai, New York, NY, USA; 2 Seaver Autism Center for Research and Treatment, Icahn School of Medicine at Mount Sinai, New York, NY, USA; 3 Department of Psychiatry and Biobehavioral Sciences, University of California, Los Angeles, CA, USA; 4 Interdepartmental Program for Neuroscience, Semel Institute for Neuroscience and Human Behavior, University of California, Los Angeles, CA, USA; 5 Semel Institute for Neuroscience and Human Behavior, University of California, Los Angeles, CA, USA; 6 Department of Psychological Science, University of Arkansas, Fayetteville, AR, USA; 7 Department of Psychology, University of California, Los Angeles, CA, USA

**Keywords:** 22q11.2, Acute stress, chronic stress, copy number variant, environmental risk factors, psychosis

## Abstract

**Background:**

The 22q11.2 deletion (22q11Del) is among the strongest known genetic risk factors for psychosis. Stress, a known risk factor for psychosis in the general population, has seldom been studied in 22q11Del. We investigated how lifetime stressors related to symptomatic outcomes in patients with 22q11Del. We also explored this association in individuals with 22q11.2 duplications (22q11Dup), which may be potentially protective against psychosis.

**Method:**

One hundred individuals (46 with 22q11Del, 30 with 22q11Dup, and 24 healthy controls; *M*_age_ = 17.30 years±10.15) were included. Logistic models were used to examine cross-sectional associations between lifetime acute and chronic stressors (severity and count) and the presence (score ⩾3) of positive, negative, and general symptoms, assessed via the Structured Interview for Psychosis-risk Syndromes (SIPS).

**Results:**

The 22q11Dup group reported the greatest number and severity of acute lifetime stressors, but did not differ from 22q11Del in chronic stressor count or severity. Lifetime chronic and acute stressors were uniquely associated with positive symptoms in 22q11Del (chronic count: odds ratio [OR] = 2.35, *p* = 0.02; chronic severity: OR = 1.88, *p* = 0.03; acute count: OR = 1.78, *p* = 0.03), but not with negative or general symptoms (*p*s > 0.05).

**Conclusion:**

Findings suggest that stress may play a role in psychotic symptoms in 22q1Del, while the 22q11Dup CNV appears protective against psychotic symptoms despite higher rates of stressors. Interventions that mitigate effects of stressors in 22qDel may reduce the odds of psychosis in this group. Prospective longitudinal research is needed to replicate these findings.

## Introduction

22q11.2 deletion syndrome (22q11Del) is a genetic disorder caused by a 1.5 to 3 Mb microdeletion on Chromosome 22, spanning up to 46 protein-coding genes (Guna, Butcher, & Bassett, 2015; Lin et al., 2020). It is among the most common genetic disorders and the most common microdeletion syndrome in humans (Kurtovic-Kozaric et al., 2016), with an incidence of approximately 1 in 4000 (Schneider et al., 2014).

Individuals with 22q11Del commonly exhibit impaired social functioning (Norkett, Lincoln, Gonzalez-Heydrich, & D'Angelo, 2017) and are at greatly elevated risk for developing psychiatric disorders across the lifespan (Bassett et al., 2005). Most notably, 22q11Del is among the strongest known genetic risk factors for schizophrenia (SCZ; Cleynen et al., 2020; Fiksinski et al., 2022), with up to 25-fold increased risk compared to the general population (Bassett & Chow, 2008; Schneider et al., 2014). A large multisite study reported the prevalence of SCZ spectrum disorders increased with age, from 10% in adolescence to 41% in adults over 25 (Schneider et al., 2014). Although neuroimaging patterns (Bagautdinova et al., 2021; Ramanathan et al., 2017) and neurocognitive factors – including executive function (Tang & Gur, 2018) and cognitive decline (Vorstman et al., 2015) – have been suggested to be predictive of SCZ in those with 22q11Del, much less is known about the role of environmental factors in the incidence of disease (Jhawar et al., 2021).

In the general population and clinical high-risk (CHR) cohorts, exposure to major life stressors is one of the strongest known environmental risk factors for SCZ and is also associated with higher rates of other psychopathology (Croft et al., 2019; Rosenberg, Lu, Mueser, Jankowski, & Cournos, 2007). In addition to childhood trauma, everyday (Norman & Malla, 1994) and lifetime stressors (Miller et al., 2001) have been associated with subsequent and increased psychotic symptoms in those with SCZ or high-risk for SCZ, respectively. Whether a specific type of stressor drives this association is unclear. Most studies have implicated early adversity or chronic life stressors, such as urban living (van Os, 2004), minority group membership (van Os, Kenis, & Rutten, 2010) racial discrimination and community violence (Rakhshan Rouhakhtar, Pitts, & Schiffman, 2019) as psychosocial risk factors for psychosis. Literature on the effects of acute stressors thus far has mostly focused on immediate physiological stress response, without a direct link to symptom severity. Some studies have found diverging results. For example, one found lower baseline cortisol levels, but no differences in cortisol response to a psychosocial stress task in first-episode psychosis patients relative to controls, as well as an association between lower baseline cortisol, fewer protective factors and higher levels of perceived stress (Seitz et al., 2019). In contrast, another study in individuals at CHR for psychosis found higher levels of cortisol associated with high subjective stress (Carol, Spencer, & Mittal, 2021). Higher baseline cortisol has also been associated with increased risk of conversion in CHR youth (Walker et al., 2013).

The effects of acute stressor exposures, although less studied in relation to SCZ, may similarly contribute to psychosis risk. While literature is limited, it has been suggested that the association between stressor exposure and psychosis might depend on stressor severity and chronicity, rather than stressor type (Croft et al., 2019). However, findings relating to the number and severity of stressors in relation to psychosis risk are mixed (e.g. Horan et al., 2005; Miller et al., 2001; Shevlin, Houston, Dorahy, and Adamson, 2008).

Research has yet to investigate whether the effects of stressors on psychosis risk in 22q11Del are similar to the associations evident in the general population and in CHR groups. Due to medical, social, and cognitive difficulties, individuals with 22q11Del are at greater risk for experiencing major life stressors, which in turn may increase their risk of psychosis (Beaton & Simon, 2011). Surprisingly, the association between stressor exposure in 22q11Del and psychosis outcomes has remained largely unexplored. One study reviewed the potential effects of chronic psychosocial stress – namely, bullying – on psychosis in 22q11Del, suggesting that stress levels may play a role in the risk of psychosis outcomes, and thus warrant further study (Mayo, Bolden, Simon, & Niendam, 2019).

To address the above-described gaps, we investigated how exposure to acute and chronic stressors occurring across the life course related to psychosis risk outcomes in patients with 22q11Del. We also explored, for the first time, the association between stress and psychosis symptoms in individuals with a duplication rather than deletion at the same locus at Chromosome 22q11.2 (22q11Dup). In contrast to 22q11Del, the 22q11Dup has demonstrated a potentially protective effect against the development of psychosis compared to 22q11Del (0% *v*. 12.3%; Lin et al., 2020), and against SCZ compared to the general population (0.014% *v.* 0.085%, OR = 0.17; Rees et al., 2014). The underlying causes for this reduced risk remain unknown. As such, we hypothesize that (1) 22q11Del will report more severe and frequent stressful life events than 22q11Dup and controls; (2) due to the increased rate of medical comorbidity in 22q11Del, we expect they will report more lifetimes stressors related to health/treatment than 22q11Dup; (3) an association between lifetime stress and positive symptoms exists in individuals with 22q11Del, but not in those with 22q11Dup; (4) the association between lifetime stressors and positive symptoms will differ between acute and chronic stressors.

A better understanding of the role of stress in 22q11Del has the potential to provide a target for preventing psychosis outcomes in this high-risk group. Furthermore, investigating the effects of acute and chronic stressor types, as well as stressor count and severity, may help to elucidate the mechanisms underlying this association, and determine which types of stressors play a major role. By focusing on multiple stressor characteristics, more targeted approaches can be developed to maximize the impact of finite resources for mitigating modifiable risk factors.

## Method

### Study population

All study participants were part of an ongoing longitudinal study of neurobehavioral profiles and brain imaging in individuals with 22q11.2 copy number variants (CNVs; 22q11Del and 22q11Dup) at the University of California at Los Angeles (UCLA; Lin et al., 2020). Recruitment, inclusion/exclusion criteria, and consent procedures are described in Participant Recruitment in the online Supplementary Material. The UCLA Institutional Review Board approved all study procedures and informed consent documents.

### Measures

#### Sociodemographic characteristics

Information regarding participants’ age, sex, race, IQ, years of completed education and highest parental education were obtained at baseline. Parental education was characterized on an ordinal scale from 0 to 9, where 0 indicates no schooling and 9 indicates completed graduate or professional school.

#### Psychosis-risk and other clinical symptoms

Supervised doctoral students in clinical psychology administered all clinical instruments. The Structured Interview for Psychosis-Risk Syndromes (SIPS; McGlashan, Miller, Woods, Hoffman, & Davidson, 2001) was administered to study participants age 10+ to identify and rate the severity of present symptoms. The SIPS consists of questions about positive symptoms, negative symptoms, general symptoms, and disorganization symptoms. Symptom severity was scored on a scale from 0 (absent) to 6 (severe). The SIPS was administered at baseline, 1 year, and 2 years follow-up. See online Supplementary Methods for information regarding psychiatric diagnoses, case consensus procedures, and assessments of psychosocial functioning.

#### Acute, chronic, and lifetime stressor exposure

Lifetime stressor exposure was assessed at baseline using the Stress and Adversity Inventory for Adults (STRAIN), an online measure developed by G. M. Slavich for systematically assessing acute and chronic stressors occurring over the entire life course and across several primary life domains and involving five different core social-psychological characteristics (Slavich & Shields, 2018; see https://www.strainsetup.com). The STRAIN consists of approximately 220 questions and assesses exposure to 55 major life stressors, including 26 acute life events and 29 chronic difficulties. Stressors are categorized into their primary life domain, coded as work, housing, education, health/treatment, reproduction, finance, interpersonal relations (parent/guardian, marital/partner, and other relationships), legal/crime, death, or life-threatening situations. Stressors are also categorized by their core social-psychological characteristic, coded as interpersonal loss, physical danger, humiliation, entrapment, and role change/disruption (i.e. a significant shift in a person's roles). See online Supplementary Methods for further details regarding STRAIN.

### Statistical analyses

Statistical analyses were performed using SAS 3.8 (Enterprise Edition) and STATA 17.

#### Baseline characteristics

Group comparisons were conducted using one-way ANOVA for continuous measures of age, IQ, years of education, and parental education. Tukey's post-hoc tests were conducted to determine pairwise differences. χ^2^ analyses were used to test for group differences in sex and race and to compare the presence of comorbid psychiatric disorders among groups. Fisher's exact *p*-values were reported for tests involving cells with fewer than five observations. Additionally, dropout analyses were conducted to examine if participants with complete data differed from those with incomplete data.

To determine baseline group differences in SIPS symptoms, weighted averages for symptom severity were calculated by symptom type (i.e. positive, negative, general, and disorganization). The weighted average ranged from 0 (all symptoms absent) to 6 (all symptoms severe). Consistent with prior publications (Jalbrzikowski et al., 2022; Weisman et al., 2017), positive, negative and general symptoms were considered ‘present’ when at least one symptom in each domain was scored as 3 (moderate) or higher at any of the three timepoints (i.e. baseline, 1 year follow-up or 2 years follow-up). A cutoff of ≥3 is standardly used to differentiate individuals with clinically significant symptoms from those without (e.g. Addington et al., 2015; Weisman et al., 2017). Due to the low numbers of disorganization symptoms endorsed in the sample, this domain was excluded from the analyses.

Differences among groups in SIPS symptom types, total lifetime acute and chronic stressor count and severity, and psychiatric diagnoses were assessed at baseline via ANOVA. Social and role functioning at baseline were also compared between groups using ANOVA.

#### Predictor models

**Fig. 1.**
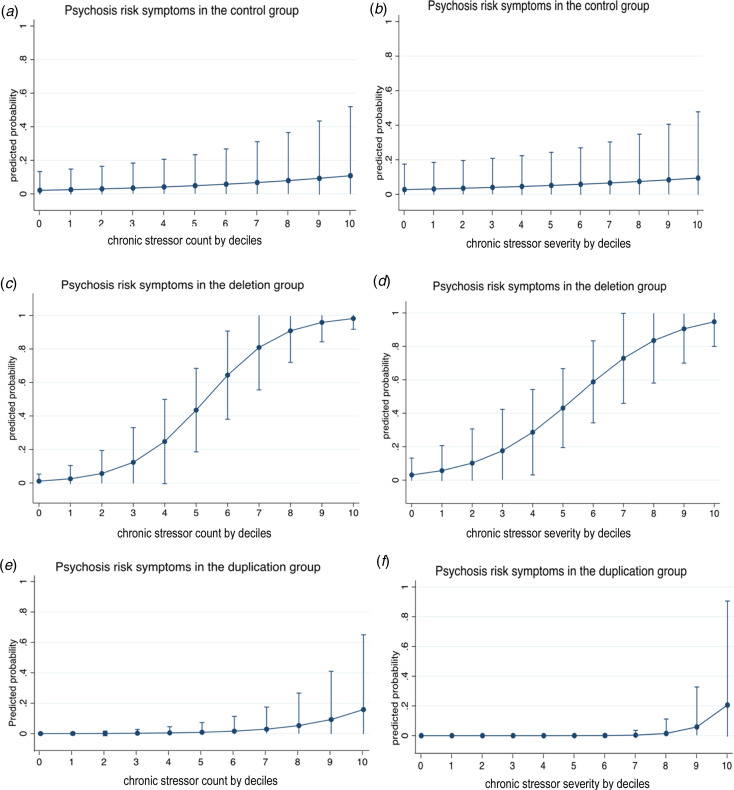
Association between an increase in chronic stress count or severity by 10% increments and positive (psychosis-risk) symptoms in the control (a, b), 22q11DS (c, d) and 22q11Dup (e, f) groups. Marginal effect estimates are drawn from logit regressions and are adjusted for age, parental education, and educational years. Interval bars represent 95% CIs.

Stratified logistic models by group were fitted to examine the association between stress and psychosis risk outcomes (yes/no). Due to the limited sample sizes, both chronic and acute stress frequencies and severity scores were divided into deciles. Plotted estimates in Fig. 1 indicate the marginal effects (predicted probabilities) following Mood (2010).

Acute lifetime stressor count, chronic lifetime stressor count, acute lifetime stressor severity, and chronic lifetime stressor severity were assessed in separate models. Age, sex, race, years of education, and parental education were included as covariates (see online Supplementary Table 1a-c for correlation matrices). A secondary analysis including only those with a diagnosed psychotic disorder was conducted controlling for age, sex, and race (education could not be controlled for due to missing data). An adjusted *p*-value threshold of 0.0125 (0.05/4 (two [acute and chronic] × two [count and severity])) was used to account for multiple comparisons.

#### Sensitivity analysis

Because stress tolerance may contribute to the stress-psychosis risk association, stress intolerance (SIPS item G4) was investigated in relation to stressor measures; it was associated with lifetime stressor exposure (including lifetime chronic stressor severity: *p* = 0.024; lifetime acute stressor count: *p* = 0.001; and lifetime acute stressor severity: *p* = 0.001), negative symptom severity (*p* < 0.001), and general symptom severity (*p* < 0.001). The main analyses were therefore repeated to additionally explore the role of stress intolerance (SIPS Item G4) in sensitivity models.

## Results

### Study population

One hundred participants were included at baseline, including 46 participants with molecularly confirmed 22q11.2 deletions (23 female; aged 6 to 42 years old) and 30 with 22q11.2 duplications (17 female; aged 6 to 49 years old). The remaining 24 participants were healthy, typically developing, age- and sex-matched controls (8 female; aged 7 to 29 years old). Thirty-five participants were excluded from analyses relating lifetime stressor exposure to psychosis-risk symptoms due to missing predictor and/or outcome measures (i.e. STRAIN and SIPS, respectively), leaving 65 participants for this analysis.

#### Dropout analysis

A dropout analysis was conducted to identify differences between participants with complete (*n* = 93) *v.* incomplete (*n* = 7) data (i.e. those with missing items on the SIPS and/or STRAIN data). Overall, those who completed all assessments were younger (*p* = 0.001) but did not differ in IQ, parental, or personal education.

### Group comparison of baseline characteristics

#### Sociodemographic characteristics

Sociodemographic characteristics can be found in Table 1. No differences in age, sex, or parental education were identified between groups. However, the groups differed with respect to race (*p* = 0.02), years of education (*p* = 0.01), IQ (*p* < 0.001) and psychiatric comorbidities (*p* < 0.001). All groups consisted of primarily White participants, with the proportion of White participants with 22q11Dup significantly greater than with 22q11Del (*p* = 0.01). The 22q11Dup group also had completed significantly fewer years of education than the 22q11Del (*p* = 0.01), but not the control group (*p* = 0.06). The 22q11Del and 22q11Dup groups were similar in IQ, but both had significantly lower IQ than controls (*p* < 0.001). See online Supplementary Material: Results and online Supplementary Table 2 for psychiatric diagnoses across groups.

**Table 1.** Sociodemographic and baseline clinical characteristics of the study sample

Sociodemographic characteristics	Control^0^ (*n* = 24)	22q11.2 Deletion^1^ (*n* = 46)	22q11.2 Duplication^2^ (*n* = 30)	Statistic
Age (years), *M* (s.d.)	16.58 (8.24)	18.37 (9.34)	16.23 (12.62)	n.s.
Female, *n* (%)	8 (33.33)	23 (50.00)	17 (56.67)	n.s.
Race (White), *n* (%)	21 (87.50)	**34 (73.91)**	**29 (96.67)**	**1 < 2**
Highest parental education, *M* (s.d.)	7.38 (1.76)	7.28 (1.41)	6.73 (1.46)	n.s
Personal education (years), *M* (s.d.)	9.75 (5.41)	**9.85 (4.86)**	**6.60 (5.28)**	**2 < 1**
IQ, *M* (s.d.)	**114.63 (14.46)**	**81.84 (13.62)**	**89.59 (18.67)**	**1,2 < 0**
Psychiatric comorbidities, *n* (%)	**5 (25.00)**	**41 (93.18)**	**26 (86.67)**	**0 < 1,2**
**Psychosocial Functioning**	***n* = 20**	***n* = 45**	***n* = 29**	
Global Assessment of Functioning (GAF), *M* (s.d.)	**88.80 (10.87)**	**53.95 (16.26)**	**54.93 (13.70)**	**1,2 < 0**
Social, *M* (s.d.)	**9.20 (0.70)**	**6.25 (2.00)**	**6.34 (1.70)**	**1,2 < 0**
Role, *M* (s.d.)	**9.25 (0.91)**	**5.56 (1.97)**	**5.76 (2.13)**	**1,2 < 0**
SIPS* symptom ratings	***n* = 18**	***n* *=* 38**	***n* = 20**	
Positive symptom severity, *M* (s.d.)	**0.78 (2.07)**	**5.63 (6.85)**	2.35 (2.87)	**0 < 1**
*Moderate or higher, n* (*%*)	**2 (10.5)**	**19 (47.5)**	6 (23.1)	**0 < 1**
Negative symptoms, *M* (s.d.)	**0.22 (0.65)**	**9.71 (7.77)**	**6.95 (5.56)**	**0 < 1,2**
*Moderate or higher, n* (*%*)	**2 (10.5)**	**31 (79.5)**	**16 (61.5)**	**0 < 1,2**
Disorganization symptoms, *M* (s.d.)	**0.38 (1.04)**	**5.00 (3.81)**	**3.10 (3.45)**	**0 < 1,2**
*Moderate or higher, n* (*%*)	**0 (0)**	**9 (23.1)**	3 (11.5)	**0 < 1**
General symptoms, *M* (s.d.)	**0.44 (1.15)**	**5.60 (3.76)**	**4.30 (3.57D)**	**0 < 1,2**
*Moderate or higher, n* (*%*)	**3 (15.8)**	**28 (71.8)**	**14 (53.8)**	**0 < 1,2**
Intolerance to stress (G4), *M* (s.d.)	**0.06 (0.24)**	**1.50 (1.62)**	**1.35 (1.39)**	**0 < 1,2**

*Note*. Bold indicates significance at *α* = 0.05. *Structured interview for Psychosis-Risk Syndromes.

#### Group differences in lifetime stressor count and severity

Group differences in lifetime stressor exposure are presented in Table 2. The 22q11Dup group reported significantly more lifetime acute stressors than controls (*p* = 0.003) but did not differ from 22q11Del. The 22q11Dup group also reported significantly greater lifetime acute stressor severity than both the 22q11Del and control group (*p* = 0.004). In contrast, 22q11Del and 22q11Dup did not differ from each other with respect to the count or severity of lifetime chronic stressors.

**Table 2.** Reported stressors across groups, as assessed with the Stress and Adversity Inventory

Stressor type	Control^0^ (*n* = 20)	22q11.2 Deletion^1^ (*n* = 33)	22q11.2 Duplication^2^ (*n* = 17)	Statistic
Stressor domain (count)				
Housing	**0.45 (0.76)**	**0.76 (1.41)**	**3.18 (3.66)**	**0,1 < 2**
Education	3.30 (3.11)	5.64 (4.51)	5.41 (4.26)	n.s.
Work	0.05 (0.22)	0.52 (1.28)	0.35 (1.00)	n.s.
Treatment/Health	**1.25 (1.48)**	2.82 (2.90)	**3.82 (3.03)**	**0 < 2**
Marital/Partner	0.60 (0.99)	0.73 (1.55)	1.00 (1.62)	n.s.
Financial	0.35 (0.81)	**0.12 (0.42)**	**1.29 (1.45)**	**1 < 2**
Legal/Crime	0.55 (1.23)	0.42 (0.79)	1.12 (2.42)	n.s.
Other relationships	1.80 (1.74)	2.03 (2.44)	2.88 (3.10)	n.s.
Guardian/Parent	1.15 (1.63)	1.21 (1.34)	1.06 (1.14)	n.s.
Death	1.05 (1.32)	1.33 (1.63)	1.59 (2.18)	n.s.
Life threatening	0.45 (0.76)	0.42 (0.97)	0.59 (0.94)	n.s.
Stressor characteristic (count)				
Interpersonal loss	3.50 (3.61)	3.06 (2.90)	4.41 (3.61)	n.s.
Physical danger	**1.15 (1.23)**	**3.15 (2.75)**	**3.94 (3.47)**	**0 < 1,2**
Humiliation	**1.95 (1.90)**	**5.09 (5.25)**	4.65 (3.66)	**0 < 1**
Entrapment	2.50 (2.44)	3.30 (2.38)	5.06 (3.93)	n.s.
Role change	**1.90 (1.83)**	**1.39 (1.64)**	**4.24 (3.17)**	**0,1 < 2**
Stressor count, *M* (s.d.)				
Acute stressor count	**10.20 (8.87)**	18.79 (15.08)	**27.29 (18.80)**	**0 < 2**
Chronic stressor count	11.80 (9.27)	13.21 (9.19)	17.29 (13.51)	n.s.
Total stressor count	**22.00 (17.53)**	32.00 (23.07)	**44.59 (29.81)**	**0 < 2**
Stressor domain (severity)				
Housing	**1.35 (2.03)**	**1.88 (2.96)**	**6.88 (6.76)**	**0,1 < 2**
Education	6.10 (6.36)	11.27 (8.97)	11.88 (8.91)	n.s.
Work	0.15 (0.67)	1.30 (3.04)	0.65 (1.90)	n.s.
Treatment/Heath	4.05 (3.35)	5.15 (6.17)	9.12 (8.53)	n.s.
Marital/Partner	1.40 (2.60)	1.76 (3.29)	2.24 (3.11)	n.s.
Financial	0.75 (2.29)	**0.09 (0.29)**	**2.70 (2.95)**	**1 < 2**
Legal/Crime	1.00 (1.92)	1.30 (2.65)	1.71 (3.53)	n.s.
Other relationships	4.65 (4.61)	4.06 (5.36)	5.88 (6.13)	n.s.
Guardian/Parent	2.95 (4.59)	2.76 (3.17)	2.94 (3.56)	n.s.
Death	2.50 (2.61)	2.45 (3.45)	3.12 (3.84)	n.s.
Life threatening	1.15 (1.93)	0.97 (2.42)	1.82 (3.26)	n.s.
Stressor characteristic (severity)				
Interpersonal loss	7.45 (6.52)	6.15 (5.95)	8.82 (7.22)	n.s.
Physical danger	3.25 (3.48)	6.58 (6.16)	8.53 (8.34)	n.s.
Humiliation	3.40 (3.07)	7.55 (8.20)	7.82 (6.60)	n.s.
Entrapment	7.2 (8.62)	9.48 (7.59)	13.82 (10.75)	n.s.
Role change	4.75 (4.47)	**3.24 (4.39)**	**9.94 (7.81)**	**1 < 2**
Stressor severity, *μ* (s.d.)				
Acute stressor severity	**17.30 (12.74)**	**25.52 (23.43)**	**44.00 (31.72)**	**0,1 < 2**
Chronic stressor severity	34.80 (32.56)	40.48 (32.98)	53.88 (42.77)	n.s.
Total stressor severity	**52.10 (43.95)**	66.00 (52.81)	**97.88 (77.35)**	**0 < 2**

*Note*. Stress and Adversity Inventory (STRAIN). Bold indicates significance at *α* = 0.0125.

Regarding group differences in the count of stressors experienced across the primary life domains and core social-psychological characteristics, the 22q11Dup group reported significantly more Housing (22q11Dup *v.* 22q11Del: Cohen *d* = 0.87, *p* = 0.008; 22q11Dup *v.* control: Cohen *d* = 1.03, *p* = 0.004:), and Role Change/Disruption (22q11Dup *v.* 22q11Del: Cohen *d* = 1.13, *p* = 0.002; 22q11Dup *v.* control: Cohen *d* = 0.90, *p* = 0.01) stressors than both the 22q11Del and control group. The 22q11Dup group also experienced more Treatment/Health (Cohen *d* = 1.08, *p* = 0.004), and Entrapment (Cohen *d* = 0.33, *p* = 0.02; though note that differences in Entrapment do not survive adjustment for multiple comparisons) than the control group, but not 22q11Del (*p* = 0.26, *p* = 0.02, *p* = 0.05, respectively). The 22q11Dup group reported more Financial lifetime stressors than 22q11Del (Cohen *d* = 1.096, *p* = 0.004), but not the control group when correcting for multiple comparisons (*p* = 0.03). Additionally, the 22q11Del group endorsed significantly more Humiliation stressors than controls (Cohen *d* = 0.80, *p* = 0.003). Both the 22q11Del and 22q11Dup groups experienced more lifetime stressors related to Physical Danger than controls (22q11Del *v.* control: Cohen *d* = 0.94 *p* < 0.001; 22q11Dup *v.* control: Cohen *d* = 1.07, *p* = 0.005).

Parallel analyses focusing on lifetime stressor severity revealed that 22q11Dup experienced more severe Housing (Cohen *d* = 0.96, *p* = 0.009) and Financial (Cohen *d* = 1.25, *p* = 0.002) lifetime stressors, as well as Role Change/Disruption (Cohen *d* = 1.06, *p* = 0.003) than the 22q11Del group. The 22q11Dup group also experienced more Housing stressors (Cohen *d* = 1.11, *p* = 0.004) than controls, but differences in lifetime stressor severity for Financial (Cohen *d* = 0.74, *p* = 0.03), Role Change/Disruption (Cohen *d* = 0.82, *p* = 0.02), and Physical Danger stressors (Cohen *d* = 0.83, *p* = 0.02) were marginal after correcting for multiple comparisons.

### Group differences in the association between lifetime stressor exposure and clinical symptoms

#### Chronic stressor exposure is associated with increased positive symptoms in 22q11Del

The associations between lifetime stressor count and severity, and symptom domains (i.e. positive, negative, and general) are presented in Table 3 (lifetime stressor count) and Table 4 (lifetime stressor severity), respectively.

**Table 3.** Lifetime stressor count and group effects on dichotomous SIPS symptom domains

Symptoms	Group	Stressor type	OR	95% CI	*p*
*Positive*	Control	Acute stressors	1.06	0.49, 2.27	0.89
		Chronic stressors	1.25	0.73, 2.14	0.41
	**Deletion**	**Acute stressors**	**1.78**	**1.05, 3.01**	**0.03**
		**Chronic stressors**	**2.35**	**1.12, 4.91**	**0.02**
	Duplication	Acute stressors	–	–	–
		Chronic stressors	1.83	0.49, 6.82	0.37
*Negative*	Control	Acute stressors	–	–	–
		Chronic stressors	0.74	0.31, 1.76	0.49
	Deletion	Acute stressors	0.99	0.69, 1.43	0.96
		Chronic stressors	0.87	0.58, 1.30	0.49
	Duplication	Acute stressors	0.85	0.49, 1.48	0.57
		Chronic stressors	0.95	0.62, 1.48	0.83
*General*	Control	Acute stressors	–	–	–
		Chronic stressors	–	–	–
	Deletion	Acute stressors	1.73	0.99, 3.01	0.054
		Chronic stressors	1.14	0.74, 1.74	0.59
	**Duplication**	**Acute stressors**	**2.07**	**1.02, 4.21**	**0.04**
		Chronic stressors	1.74	0.98, 3.10	0.058

*Note*. Logistic regression model odds ratios and parameter estimates for psychosis risk where one or more symptoms are rated 3 or greater in severity. Bold indicates significance at *α* = 0.05.

**Table 4.** Lifetime stressor severity and group effects on dichotomous SIPS symptom domains

Symptoms	Group	Stressor type	*OR*	*95% CI*	*p*
*Positive*	Control	Acute stressors	*1*.*49*	*0.64, 3.47*	*0*.*35*
		Chronic stressors	*1*.*21*	*0.66, 2.23*	*0*.*61*
	Deletion	Acute stressors	1.30	0.90, 1.87	0.17
		**Chronic stressors**	**1.88**	**1.03, 3.45**	**0.04**
	Duplication	Acute stressors	–	–	–
		Chronic stressors	4.17	0.25, 68.72	0.32
*Negative*	Control	Acute stressors	0.79	0.31, 2.00	0.61
		Chronic stressors	0.70	0.26, 1.85	0.47
	Deletion	Acute stressors	0.90	0.62, 1.30	0.57
		Chronic stressors	1.01	0.68, 1.50	0.97
	Duplication	Acute stressors	0.96	0.60, 1.52	0.85
		Chronic stressors	1.02	0.67, 1.55	0.93
*General*	Control	Acute stressors	–	–	–
		Chronic stressors	–	–	–
	Deletion	Acute stressors	1.56	0.92, 2.64	0.10
		Chronic stressors	1.47	0.92, 2.35	0.11
	Duplication	Acute stressors	1.51	0.95, 2.40	0.08
		**Chronic stressors**	**1.71**	**1.01, 2.90**	**0.047**

*Note*. Logistic regression model odds ratios and parameter estimates for psychosis risk where one or more symptoms are rated 3 or greater in severity. The integration of environmental factors and EMA analytic plan. Bold indicates significance at *α* = 0.05.

Figure 1 plots the association between by decile increments in chronic stressor count (a) or severity (b) and positive symptoms in the control, 22q11Del and 22q11Dup groups. See online Supplementary Figure 1 for association between continuous chronic stress count or severity and positive symptoms.

Although lifetime chronic stressor exposure and severity were similar in the 22q11Del and 22q11Dup groups, chronic stressor count and severity were each only associated with the presence of positive symptoms in 22q11Del, as hypothesized (count: OR = 2.35, 95% CI [1.12–4.91], *p* = 0.02; severity: OR = 1.88, 95% CI [1.03–3.45], *p* = 0.04). The results were similar when controlling for IQ rather than years of education (count: OR = 2.70, 95% CI [1.19–6.13], *p* = 0.02; severity: OR = 2.08, 95% CI [1.05–4.15], *p* = 0.04; see online Supplementary Table 3).

Additionally, the count (but not severity, OR = 1.30, 95% CI [0.90–1.87], *p* = 0.17) of acute stressors was marginally associated with the presence of positive symptoms in those with 22q11Del (OR = 1.78, 95% CI [1.05–3.01], *p* = 0.03), but not 22q11Dup (*p*-value could not be calculated due to low numbers of individuals with outcome of interest). The addition of SIPS Item G4 (impaired stress tolerance) in the model did not affect the results. However, when controlling for IQ in place of years of education, the association in 22q11Del is reduced to a trend level (OR = 1.50, 95% CI [0.96–2.36], *p* = 0.08; see online Supplementary Table 3).

In 22q11Dup, the association between lifetime acute and chronic stressors with the presence of general symptoms was both trending toward significance. Specifically, lifetime acute stressor count (OR = 2.07, *p* = 0.04) and lifetime chronic stressor severity (OR = 1.71, *p* = 0.047) were both marginally associated with an increased risk of general symptoms in the 22q11Dup group. Neither chronic nor acute stressor exposure was related to negative symptoms in any of the groups (*p*s > 0.05).

### Secondary analyses in 22qDel with psychotic disorder

Among those with a diagnosed psychotic disorder, given the small sample, the associations between stressor count and severity and psychotic disorder were not statistically significant, but had large effect sizes (chronic count: OR = 3.53, 95% CI: [0.97–12.94], *p* = 0.057; chronic severity: OR = 2.70, 95% CI: [ 0.96–7.55], *p* = 0.059; acute count: OR = 1.28, 95% CI: [0.78–2.13], *p* = 0.33; acute severity: OR = 0.99, 95% CI: [ 0.63–1.57], *p* = 0.963), trending toward significance for chronic stressors in the 22q11Del group. The association between lifetime stressors and the presence of a psychotic disorder could not be calculated in the control group or 22q11Dup group due to the absence of diagnosed psychotic disorders (*n* = 0; see online Supplementary Table 2).

### Group differences in the association between lifetime stressors and functioning

Regression analyses did not reveal any significant associations between lifetime acute or chronic stressor exposure and baseline GAF, GFS or GFR scores across groups (*p*s > 0.05).

## Discussion

To our knowledge, this study is the first to assess differences in exposure to stressors across the life course between individuals with reciprocal 22q11.2 CNVs and healthy controls. Contrary to expectations, we found that individuals with 22q11Dup experienced the most lifetime stressors compared to those with 22q11Del and the control group. Specifically, individuals with 22q11Dup experienced greater lifetime stressor exposure and severity for stressors involving housing, finance, role change, and physical danger. Despite reporting significantly more environmental stressors, individuals with 22q11Dup did not endorse more SIPS symptoms than those with 22q11Del. We also examined the association between lifetime stressors and clinical symptom expression in this sample of 22q11.2 CNV carriers. Consistent with our hypothesis, we found that chronic and acute stressors were related to positive symptoms in individuals with 22q11Del but not those with 22q11Dup. Interestingly, this difference was not explained by differences in stress tolerance. Instead, both acute stressor frequency and chronic stressor severity were marginally associated with general symptom severity (e.g. mood, sleep disturbance) in the 22q11Dup group.

Notably, the associations between lifetime stressors and SIPS symptoms observed in the deletion group were specific to positive symptoms and most robust for chronic stressors. It is well-established that stressful experiences are related to psychosis outcomes in the general population, particularly chronic early life stressors (Stanton, Denietolis, Goodwin, & Dvir, 2020). Many studies have demonstrated associations between stress and psychopathology and provided biological evidence for the role of stress in SCZ (Pruessner, Cullen, Aas, & Walker, 2017). Therefore, it is unsurprising that among individuals with psychotic disorders, stress is highly prevalent (Croft et al., 2019). The findings in this sample suggest that chronic life stressors may be an important risk factor to target in this population.

Consistent with prior research, 22q11Del patients showed elevated symptom levels across domains (Lin et al., 2020). Interestingly, the association between stress and positive symptoms within individuals with 22q11Del was robust even when controlling for subjective stress tolerance, suggesting that while stress may play a role, it is not the only differentiating factor for psychosis outcomes in 22q11Del *v.* 22q11Dup.

Stressors occurring across the life course were not associated with negative or general symptom outcomes in 22q11Del, suggesting that other factors may play a greater role in the presence and severity of these symptoms in this group. In contrast, we did detect an association between lifetime stressors and general symptoms in the 22q11Dup group, suggesting that life stressors may have a different role in symptomatology in reciprocal 22q11.2 CNVs.

Apart from psychotic disorders, individuals with both 22q11.2 CNVs share similar risk for many comorbid conditions and other neuropsychiatric disorders (e.g. autism spectrum disorder, ADHD; Beaton and Simon, 2011). Our study affirms similar risks of various psychiatric disorders and found elevated rates of negative, disorganized, and general symptoms both 22q11.2 CNV groups. In contrast to 22q11Del, lifetime stressors were only related to the presence of general symptoms in 22q11Dup, and not positive psychosis-risk symptoms. Despite experiencing significantly higher levels of acute and chronic stressors, no study participants with 22q11Dup were diagnosed with a psychotic disorder, whereas 16% (*n* = 7) of the deletion group had this diagnosis. This result is consistent with prior research suggesting that 22q11Dup may be potentially protective against the development of psychotic disorders (Lin et al., 2020; Rees et al., 2014). This may suggest that those with 22q11Dup do not respond to stressors in the same way as those with 22q11Del with respect to psychotic-like (positive) symptoms, but may be more likely to have non-specific (general) symptoms in response to stressors. Interestingly, while both 22q11.2 CNVs put individuals at considerable risk for several health problems and, consequently, related stressors, there were several differences in reported stress groups. The 22q11Dup group reported the most stressful events overall, both in count and severity, mostly accounted for by higher levels of stress related to housing, finance, and role change. The causes of these differences are unclear but may relate to different patterns of inheritance between 22q11Del and 22q11Dup. Specifically, the 22q11Dup tends to be familial (Armando et al., 2018) while up to 90% of 22q11.2 deletions occur de novo (International 22q11.2 Foundation, 2022). Parents with health problems and disabilities hold a unique role both as caregivers and the receivers of care. In addition to caregiving burden, they are tasked with managing their own health problems and disabilities (Parchomiuk, 2016). Importantly, parents with disabilities, differ in employment and income, being more likely to be unemployed and earning, on average, $ 15 000 lower income (Olkin, Abrams, Preston, & Kirshbaum, 2006). Consequently, the effects of these stressors are often seen across generations (e.g. generational poverty). Notably, reported stress related to health and treatment was similar between the 22q11.2 CNV groups, further suggesting a larger role of family life and psychosocial difficulties unrelated to health problems for individuals with 22q11Dup. Exploring the role of the home environment in relation to stressful experiences in 22q11 CNV carriers goes beyond the scope of this study but could be the subject of further investigation.

This study is the first to examine the effects of both acute and chronic lifetime stressors on clinical symptoms in individuals with 22q11.2 CNVs. Our findings offer new evidence for the role of environmental factors in the expression of psychosis risk in a genetically vulnerable population, as well as potentially protective effects of the 22q11.2 duplication, a novel area of research.

An important strength of the study was the use of the STRAIN. Developed in response to a dearth of research assessing lifetime stressor exposure (Slavich, 2019), the STRAIN is a reliable, user-friendly instrument that can be completed in less than 20 min (Slavich & Shields, 2018). Additionally, despite requiring participants to recall past stressors, completing the STRAIN is not associated with increases in negative moods, and has been found to be insensitive to personality styles and social desirability, both of which can affect reporting (Slavich & Shields, 2018). The STRAIN has been well-validated in relation to a number of cognitive, behavioral, and clinical outcomes (Cazassa, Oliveira, Spahr, Shields, & Slavich, 2020), and has high test-retest reliability (e.g. *r*_icc_ = 0.953). However, the STRAIN primarily focuses on individual-level stressors and future studies are encouraged to consider a broader perspective using additional measures that address how systemic issues may potentially contribute to more lifetime stress events.

Several limitations must also be noted. First, there is a risk of ascertainment bias since participants with 22q11.2 CNVs were recruited from support groups and medical or genetics clinics, excluding those who were undiagnosed or without access to medical care. There is also the possibility of unaccounted heterogeneity in the clinical samples, since the size and breakpoints of the 22q11.2 CNV were not considered. It is plausible that these characteristics of the 22q11.2 mutation could impact the type and/or severity of disorder(s) or symptoms experienced.

Furthermore, there are challenges associated with subjective, retrospective self-report measures, which are vulnerable to recall biases and the natural decay of human memory. In addition, many individuals in the study experienced several psychiatric symptoms that may impair accurate recall. Another challenge related to the STRAIN is that stressful events related to sensitive topics may have been underreported due to repressed memories, desire to protect existing interpersonal relationships, or social desirability.

Limitations to the study design and sample size further restricted which analyses could be run and the interpretation of the findings. For example, the cross-sectional design does not allow for causation to be established. Because existing symptoms may contribute to stressful events and vice versa, the directionality of the relationship is unclear, and is likely complex and bidirectional. Longitudinal follow-up studies are underway to better address these complex relationships over time. Small sample size also limited the ability to perform certain analyses (e.g. on disorganized symptoms) and likely underpowered others. Outcomes were dichotomized as a result, but this may have caused some effects to be missed.

In conclusion, the findings affirm that stress is a relevant risk factor for psychosis outcomes, particularly in the 22q11Del group. These results tentatively suggest that interventions focused on minimizing the effects of stressors may help to reduce risk for psychosis in this high-risk group. Moreover, individuals with 22q11Dup reported general symptoms but did not present with psychotic disorders, nor any association between stressful events and psychosis-risk symptoms despite having more stressful events experienced. Longitudinal research is needed to replicate our results and establish temporal precedence. Additionally, further research is required to identify other factors that may explain why 22q11Del is at considerably high risk for psychosis while 22q11Dup is not. Our findings offer new evidence for the role of environmental factors in the expression of psychosis risk in a genetically vulnerable population, as well as potentially protective effects of the 22q11Dup, a novel area of research.

## Supporting information

Modasi et al. supplementary material Modasi et al. supplementary material

## Supplementary material

The supplementary material for this article can be found at https://doi.org/10.1017/S0033291723000740.
