# Supplementary material for: Associations between acute and chronic lifetime stressors and psychosis-risk symptoms in individuals with 22q11.2 copy number variants
Source: Psychol Med. 2023 Apr 20;53(15):7222–31. doi: 10.1017/S0033291723000740 (PMC10719673; doi:10.1017/S0033291723000740)
Supplement: Modasi et al. supplementary material [file S0033291723000740sup001.docx]

**Supplemental Material**

**Methods**

**Participant recruitment.** Participants were recruited from support groups or medical or genetics clinics. Typically developing and demographically comparable control participants were recruited through online advertisements and flyers in local community sites. Written and oral informed consent was obtained from all participants. Written assent was obtained from those under 18 with written consent from a parent or guardian. Participants with a neurological or medical condition (unrelated to their genetic disorder) that affects brain structure and/or function were excluded. Other exclusion criteria were a history of head injury with loss of consciousness, insufficient fluency in English, and/or substance or alcohol use disorder within the past 6 months. The UCLA Institutional Review Board approved all study procedures and informed consent documents.

**Stress and Adversity Inventory for Adults (STRAIN).** In the STRAIN, stressors are defined by the potential to be enduring, such that acute stressors typically occur over 1 to 2 days (e.g., death, initiation of divorce, job loss), and chronic stressors are more likely to be persistent (e.g., unstable housing, financial difficulties, relationship problems). For each stressor, participants are asked to rate the count, severity, duration, and timing of the stressors endorsed. For the present study, the STRAIN’s primary outcomes were used to create the following variables: lifetime acute stressor count, lifetime chronic stressor count, total lifetime stressor count, lifetime acute stressor severity, lifetime chronic stressor severity, and total lifetime stressor severity. Higher scores represent greater stressor exposure (i.e., for stressor count variables) or greater cumulative stress severity (i.e., for stressor severity variables).

**Psychiatric diagnoses and comorbidities.** Psychiatric diagnoses and comorbidities were assessed using the Structured Clinical Interview for DSM-5 Disorders (SCID-5) with an additional developmental disorders module (First, Williams, Karg, & Spitzer, 2015). Participants aged 10 and under were assessed using the Computerized Diagnostic Interview Schedule for Children (C-DISC). All diagnoses were determined by trained Masters’ or PhD-level clinicians who participated in an ongoing quality assurance program (e.g., Lin et al., 2020). Training, reliability, and ongoing quality assurance procedures for structured interview assessments and rating scales are described elsewhere (Jalbrzikowski et al., 2013; Jalbrzikowski et al., 2017).

**Psychosocial functioning.** The Global Functioning: Role (GFR) and Global Functioning: Social (GFS) scales were used to rate age-specific role and social functioning, respectively, ranging from 1 (extreme dysfunction) to 10 (superior functioning; Cornblatt et al., 2007). The GFR and GFS scales have been shown to have adequate construct reliability and result in high interrater reliability (Cornblatt et al., 2007). The GFR assesses functioning based on level of independence, and academic and occupational performance. The GFS examines the quality and quantity of interpersonal relationships, interpersonal conflict, and family involvement. The lowest level of functioning in the past month (i.e., the current functioning score) was considered at baseline.

**Results**

**Comorbidities.** The breakdown of diagnoses by disorder type is detailed in Supplemental Table 2. The presence of psychiatric comorbidities (autism spectrum disorder, ADHD, anxiety disorder, and other disorders) among both the 22q11Del and 22q11Dup groups was significantly greater than for controls (*p* < .001) but did not differ from each other. Notably, in 22q11Del, 16% (*n* = 7) were diagnosed with a psychotic disorder as compared to none in the 22q11Dup or control groups. Differences in reported SIPS symptoms have previously been reported in Lin et al. (2020).

Supplemental Table 1: Correlation Matrix of Study Measures.

a) Correlation matrix of study measures within healthy controls


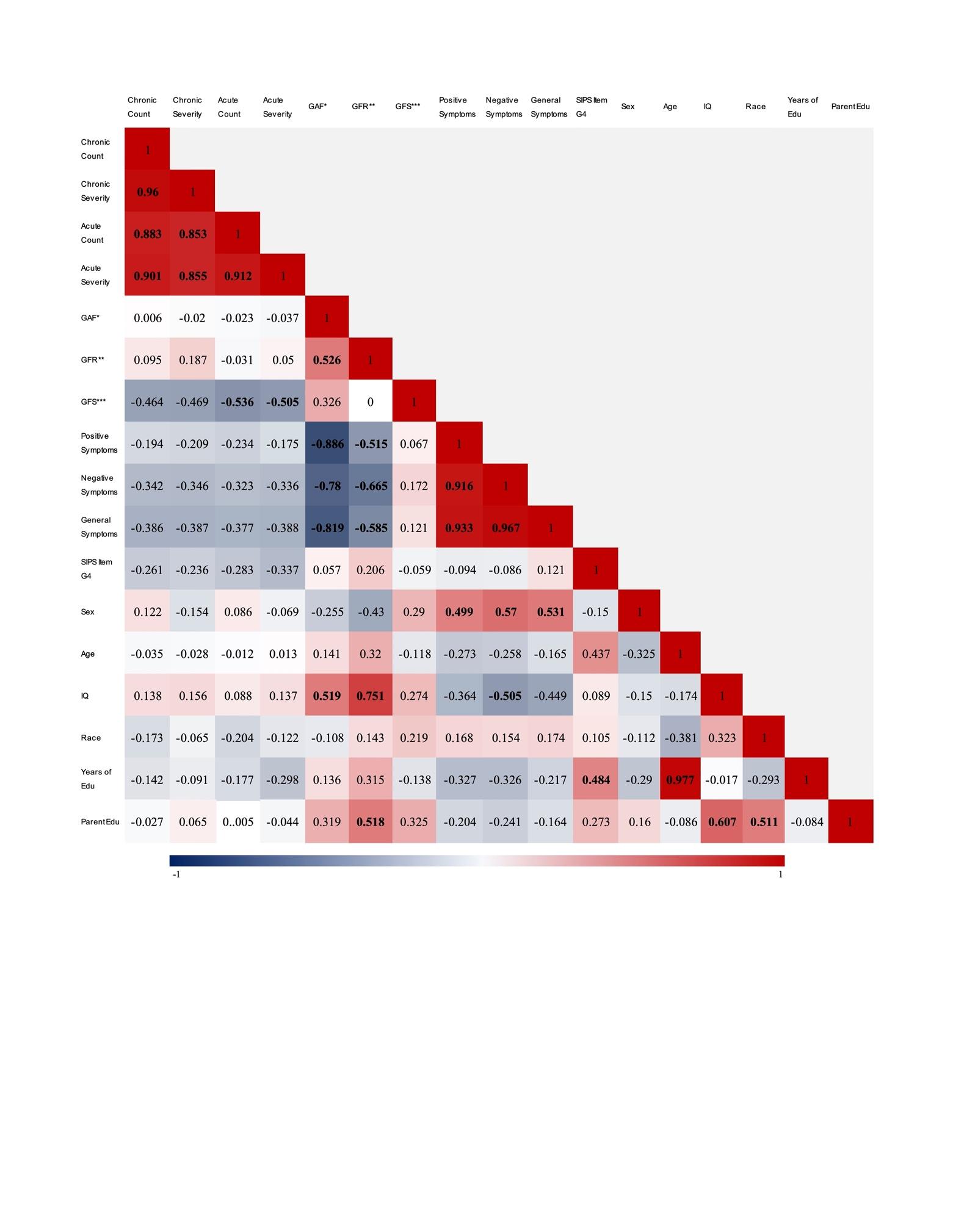


b) Correlation matrix of study measures within the 22q11Del group


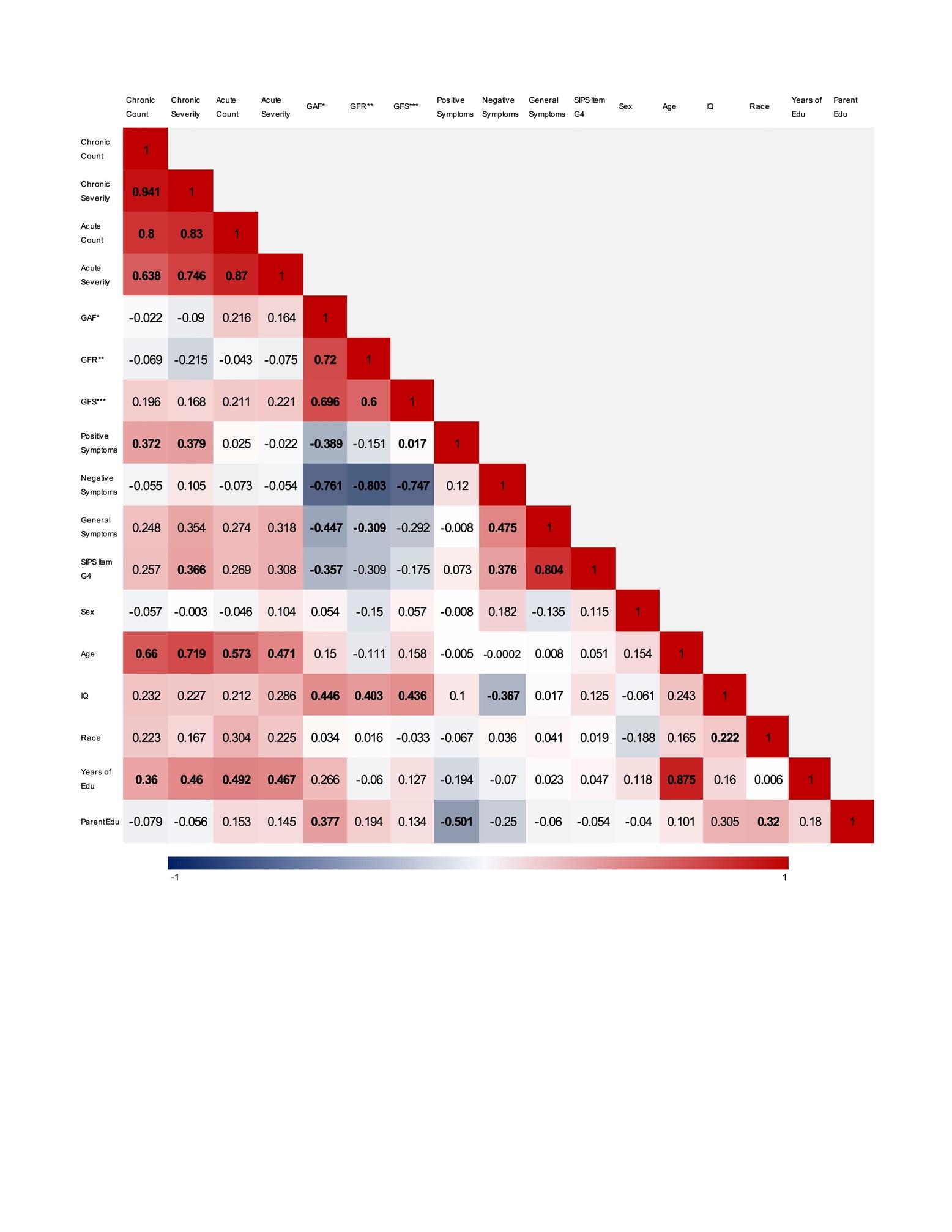


c) Correlation matrix of study measures within the 22q11Dup group


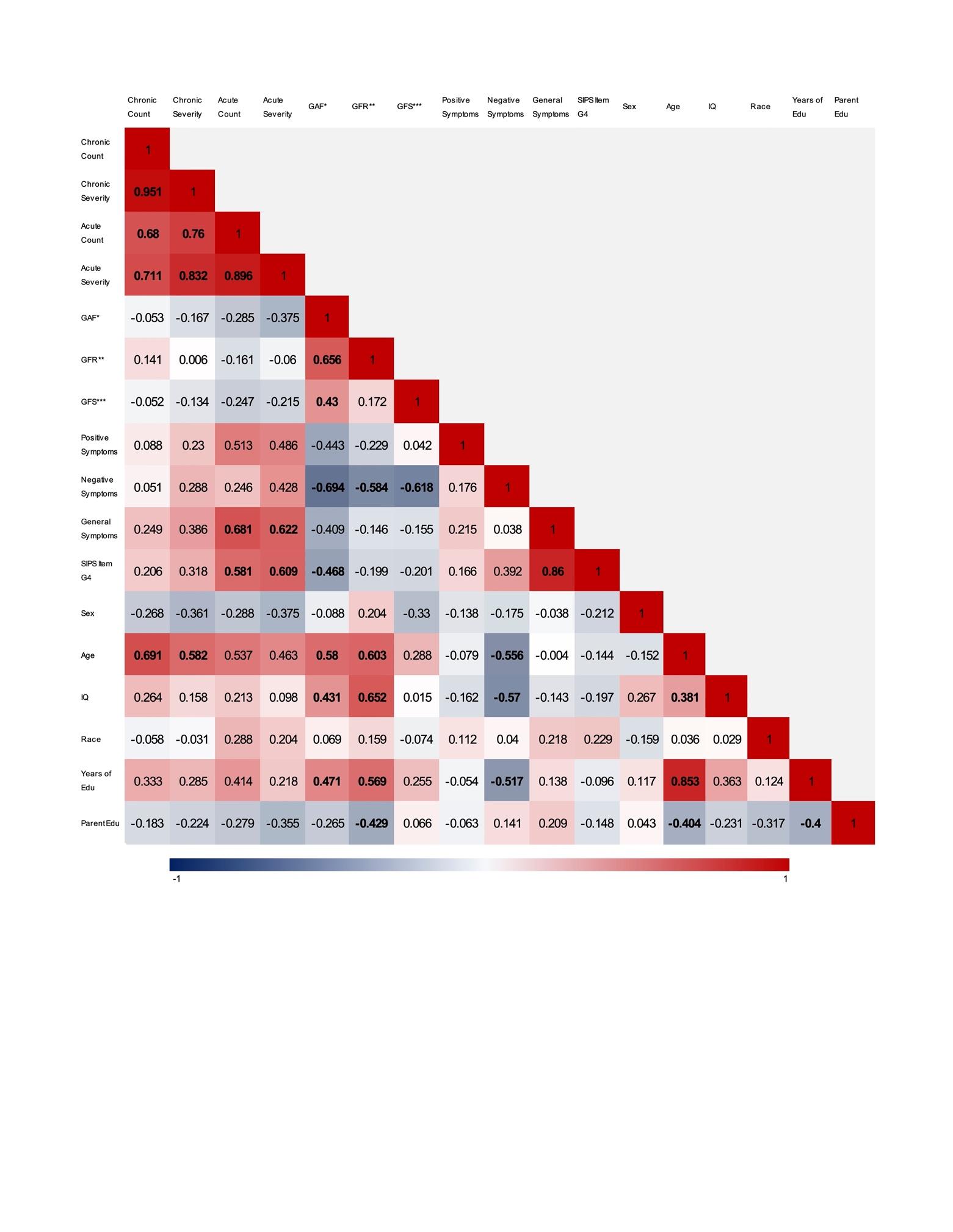


Note. a) Control group; b) 22q11Del group; c) 22q11Dup group. Colors indicate strength and direction of correlation. SIPS Item G4 = Impaired Tolerance to Normal Stress; *GAF = Global Assessment of Functioning; **GFR = Global Functioning: Role; ***GFS = Global Functioning: Social. Bold indicates significance at α =.05.

Supplemental Table 2

| **Supplemental Table 2.** Comorbid Psychiatric Diagnoses at Baseline | | | | |
| --- | --- | --- | --- | --- |
| **Diagnosis** | **Control^0^** | **22q11.2 deletion^1^** | **22q11.2 duplication^2^** | **Statistic** |
| Psychotic disorder, n (%) | 0 (0) | 7 (15.91) | 0 (0) | _ |
| Autism spectrum disorder, n (%) | **0 (0)** | **15 (34.09)** | **11 (36.67)** | **0<1,2** |
| Mood disorder, n (%) | 1 (4.76) | 11 (25.00) | 4 (13.33) | n.s. |
| Anxiety disorder, n (%) | **0 (0)** | **23 (52.27)** | **15 (50.00)** | **0<1,2** |
| ADHD, n (%) | **2 (9.52)** | **24 (54.55)** | **11 (36.67)** | **0<1,2** |
| Other disorder, n (%) | **2 (9.52)** | **16 (36.36)** | **15 (50.00)** | **0<1,2** |

Note. Diagnoses were assessed using the SCID/CDISC. ASD was assessed via the ADI/ADOS. Bold indicates significance at α = .05

Supplemental Table 3

| **Supplemental Table 3a.** Lifetime Stressor Count and Group Effects on Dichotomous SIPS Symptom Domains | | | | | | | | |  |
| --- | --- | --- | --- | --- | --- | --- | --- | --- | --- |
|  |  |  | ***Controlling for Years of Education*** | | | ***Controlling for IQ*** | | | |
| **Symptoms** | **Group** | **Stressor Type** | **OR** | **95% CI** | ***p*** | **OR** | ***95% CI*** | ***p*** |  |
| *Positive* | Control | Acute stressors | 1.06 | 0.49, 2.27 | 0.89 |  |  |  |  |
|  |  | Chronic stressors | 1.25 | 0.73, 2.14 | 0.41 |  |  |  |  |
|  | Deletion | Acute stressors | **1.78** | **1.05, 3.01** | **0.03** | 1.50 | 0.96, 2.36 | 0.08 |  |
|  |  | Chronic stressors | **2.35** | **1.12, 4.91** | **0.02** | **2.70** | **1.19, 6.13** | **0.02** |  |
|  | Duplication | Acute stressors | _ | _ | _ |  |  |  |  |
|  |  | Chronic stressors | 1.83 | 0.49, 6.82 | 0.37 |  |  |  |  |
| *Negative* | Control | Acute stressors | _ | _ | _ |  |  |  |  |
|  |  | Chronic stressors | 0.74 | 0.31, 1.76 | 0.49 |  |  |  |  |
|  | Deletion | Acute stressors | 0.99 | 0.69, 1.43 | 0.96 |  |  |  |  |
|  |  | Chronic stressors | 0.87 | 0.58, 1.30 | 0.49 |  |  |  |  |
|  | Duplication | Acute stressors | 0.85 | 0.49, 1.48 | 0.57 |  |  |  |  |
|  |  | Chronic stressors | 0.95 | 0.62, 1.48 | 0.83 |  |  |  |  |
| *General* | Control | Acute stressors | _ | _ | _ |  |  |  |  |
|  |  | Chronic stressors | _ | _ | _ |  |  |  |  |
|  | Deletion | Acute stressors | 1.73 | 0.99, 3.01 | 0.054 |  |  |  |  |
|  |  | Chronic stressors | 1.14 | 0.74, 1.74 | 0.59 |  |  |  |  |
|  | Duplication | Acute stressors | **2.07** | **1.02, 4.21** | **0.04** | 2.08 | 0.97, 4.46 | 0.06 |  |
|  |  | Chronic stressors | 1.74 | 0.98, 3.10 | 0.058 |  |  |  |  |

| **Supplemental Table 3a.** Lifetime Stressor Severity and Group Effects on Dichotomous SIPS Symptom Domains | | | | | | | | |  |
| --- | --- | --- | --- | --- | --- | --- | --- | --- | --- |
|  |  |  | ***Controlling for Years of Education*** | | | ***Controlling for IQ*** | | | |
| **Symptoms** | **Group** | **Stressor Type** | **OR** | **95% CI** | ***p*** | **OR** | ***95% CI*** | ***p*** |  |
| *Positive* | Control | Acute stressors | *1.49* | *0.64, 3.47* | *0.35* |  |  |  |  |
|  |  | Chronic stressors | *1.21* | *0.66, 2.23* | *0.61* |  |  |  |  |
|  | Deletion | Acute stressors | 1.30 | 0.90, 1.87 | 0.17 |  |  |  |  |
|  |  | Chronic stressors | **1.88** | **1.03, 3.45** | **0.04** | **2.08** | **1.05, 4.15** | **0.04** |  |
|  | Duplication | Acute stressors | _ | _ | _ |  |  |  |  |
|  |  | Chronic stressors | 4.17 | 0.25, 68.72 | 0.32 |  |  |  |  |
| *Negative* | Control | Acute stressors | 0.79 | 0.31, 2.00 | 0.61 |  |  |  |  |
|  |  | Chronic stressors | 0.70 | 0.26, 1.85 | 0.47 |  |  |  |  |
|  | Deletion | Acute stressors | 0.90 | 0.62, 1.30 | 0.57 |  |  |  |  |
|  |  | Chronic stressors | 1.01 | 0.68, 1.50 | 0.97 |  |  |  |  |
|  | Duplication | Acute stressors | 0.96 | 0.60, 1.52 | 0.85 |  |  |  |  |
|  |  | Chronic stressors | 1.02 | 0.67, 1.55 | 0.93 |  |  |  |  |
| *General* | Control | Acute stressors | _ | _ | _ |  |  |  |  |
|  |  | Chronic stressors | _ | _ | _ |  |  |  |  |
|  | Deletion | Acute stressors | 1.56 | 0.92, 2.64 | 0.10 |  |  |  |  |
|  |  | Chronic stressors | 1.47 | 0.92, 2.35 | 0.11 |  |  |  |  |
|  | Duplication | Acute stressors | 1.51 | 0.95, 2.40 | 0.08 |  |  |  |  |
|  |  | Chronic stressors | **1.71** | **1.01, 2.90** | **0.047** | 3.03 | 0.54, 16.96 | 0.208 |  |

Note. Bold indicates significance at α = .05

Supplemental Figure 1: Association between increased continuously measured chronic lifetime stress count or severity scores and positive (psychosis-risk) symptoms in the control (a, b), 22q11DS (c, d) and 22q11Dup (e, f) groups.

**
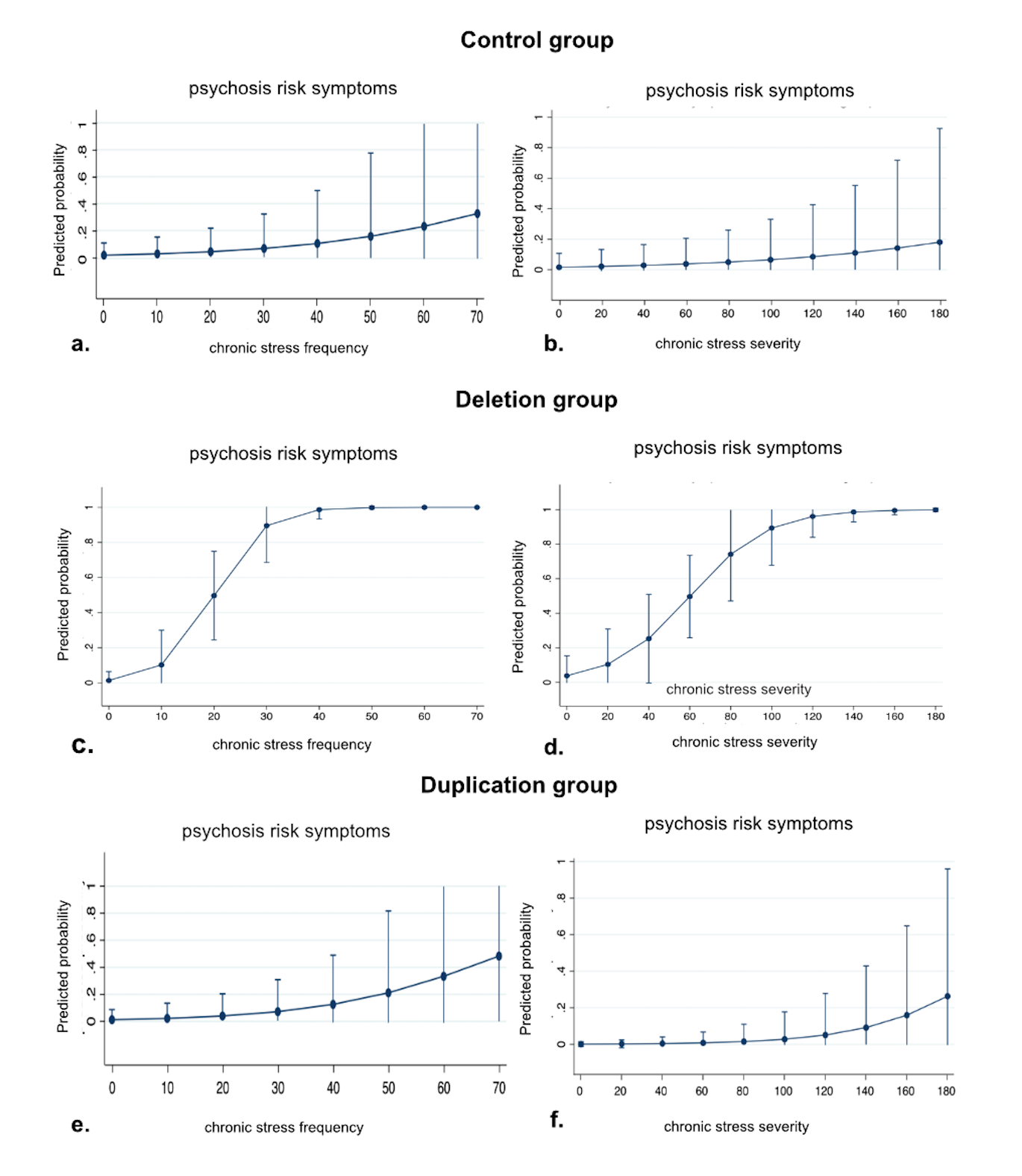
**

**References**

Cornblatt, B. A., Auther, A. M., Niendam, T., Smith, C. W., Zinberg, J., Bearden, C. E., & Cannon, T. D. (2007). Preliminary findings for two new measures of social and role functioning in the prodromal phase of schizophrenia. Schizophrenia Bulletin, 33(3), 688–702. https://doi.org/10.1093/schbul/sbm029

First, M. B., Williams, J. B. W., Karg, R. S. & Spitzer, R. L. (2015). Structured clinical interview for DSM-5—Research version (SCID-5 for DSM-5, Research Version; SCID-5-RV). Arlington, VA, American Psychiatric Association.

Jalbrzikowski, M., Ahmed, K. H., Patel, A., Jonas, R., Kushan, L., Chow, C., & Bearden, C. E. (2017). Categorical versus dimensional approaches to autism-associated intermediate phenotypes in 22q11. 2 microdeletion syndrome. Biological Psychiatry: Cognitive Neuroscience and Neuroimaging, 2(1), 53-65. https://doi.org/10.1016/j.bpsc.2016.06.007

Jalbrzikowski, M., Jonas, R., Senturk, D., Patel, A., Chow, C., Green, M. F., & Bearden, C. E. (2013). Structural abnormalities in cortical volume, thickness, and surface area in 22q11. 2 microdeletion syndrome: relationship with psychotic symptoms. NeuroImage: Clinical, 3, 405-415. https://doi.org/10.1016/j.nicl.2013.09.013

Lin, A., Vajdi, A., Kushan-Wells, L., Helleman, G., Hansen, L. P., Jonas, R. K., … Bearden, C. E. (2020). Reciprocal copy number variations at 22q11.2 produce distinct and convergent neurobehavioral impairments relevant for schizophrenia and autism spectrum disorder. Biological Psychiatry, 88(3), 260–272. https://doi.org/10.1016/j.biopsych.2019.12.028
